# Successful treatment of a case with NUT midline carcinoma in the larynx and review of the literature

**DOI:** 10.1002/ccr3.2568

**Published:** 2019-11-28

**Authors:** Hai Zhang, Ming‐Hua Liu, Jie Zhang, Shu‐Peng Luo, Chao‐Bo Wang, Zeng‐Hu Zhao, Yan‐Li Ge, Jian‐Yu Zhang, Feng‐Hao Geng

**Affiliations:** ^1^ Department of Oncology The Hospital of 81st Group Army PLA Zhang Jia Kou China; ^2^ Department of Pharmacy The Hospital of 81st Group Army PLA Zhang Jia Kou China; ^3^ Department of Radiation Medicine Faculty of Preventive Medicine Air Force Medical University Xi'an China; ^4^ Outpatient Department of Dongcheng Fourth Cadres'Sanatorium Beijing China

**Keywords:** intensity‐modulated radiation therapy, larynx, NUT midline carcinoma, successful treatment, traditional Chinese medicine

## Abstract

In this report, we gave the first case of successful treatment for laryngeal NMC, which is exceedingly rare with dismal prognosis. intensity‐modulated radiation therapy accompanied by traditional Chinese medicine was administrated for the young woman, instead of radical resection, and she got continuous remission for more than 2 years, with no recurrence detected.

## Graphical abstract

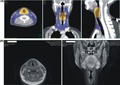

## 1 INTRODUCTION

Nuclear protein in testis (NUT) midline carcinoma (NMC) is a rare and highly aggressive cancer,^1^ frequently accompanied by distant metastases, and commonly arises from the midline structures, such as head, neck, and thorax. For frequently undiagnosed or misdiagnosed, the prevalence is still unknown and by the end of 2017, the largest pool of NMC patients from reported meta‐analysis contained only 119 cases, worldwide.^2^

NUT midline carcinoma that arises in the larynx is extremely rare, with only seven cases reported thus far. All the seven cases presented at an extensive clinical stage and showed a poor prognosis with the survival time ranged from 3 to 11 months.^3^, ^4^, ^5^, ^6^

We present the first case of a laryngeal NMC patient treated with intensity‐modulated radiation therapy (IMRT) and traditional Chinese medicine (TCM) after local resection, which exhibits long‐time survival potential.

## 2 CASE PRESENTATION

**Figure 1:**
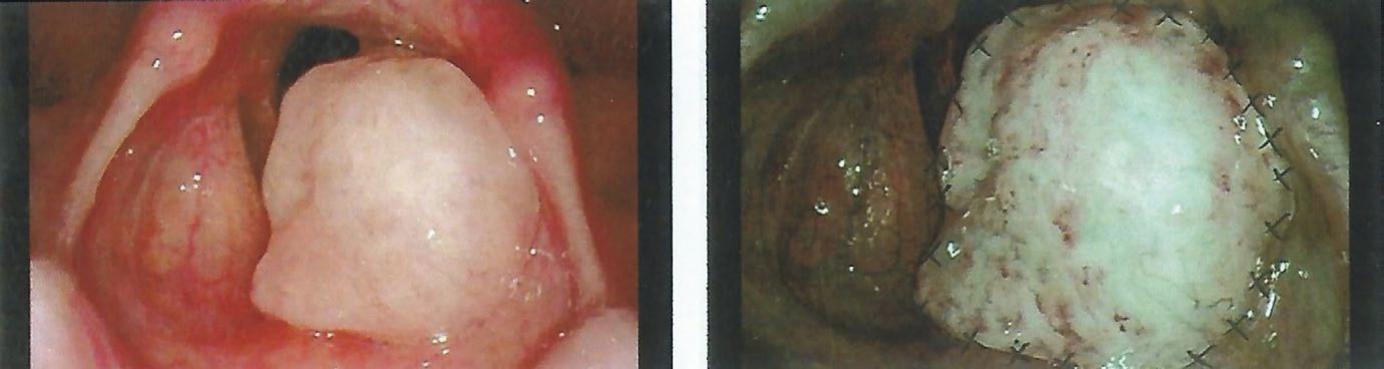
Laryngoscope examination of the patient at initial consultation: possible angiogenesis in the laryngeal tumor

A 20‐year‐old woman initially presented with hoarseness in June 2016 and developed symptoms of pain, itching and foreign body sensation in the pharynx and hacking cough in the last 2 months. She was never a smoker and denied any medical history. For a definitive diagnosis and treatment, she turned to Beijing Tong Ren Hospital in October. Laryngoscope examination revealed a space‐occupying lesion in the vallecula of epiglottis (Figure 1) and then through transoral CO2 laser microsurgical resection under general anesthesia, a mass of 1 × 1.5 cm in the left vocal cord was excised. Frozen section analysis predicted malignancy with poorly differentiated cells, which in favor of the possibility of mixed neuroendocrine carcinoma. Then, local resection was performed to remove the whole left vocal cord from the end of thyroid cartilage. Histology of excision showed that the tumor was around 1.6 × 1.5 × 0.7 cm and cells were grossly consisted of sheets of immature cells with abrupt of keratinization, which was morphologically typical for NUT midline carcinoma. Meanwhile, tumor cells were not detected in incised margin and immune‐reactive for P53 (++), Ki‐67 (index for 40%), CK+, CgA (−), Syn (−), CD56 (−), NSE (−), and P40 (+) by IHC analysis.

**Figure 2:**
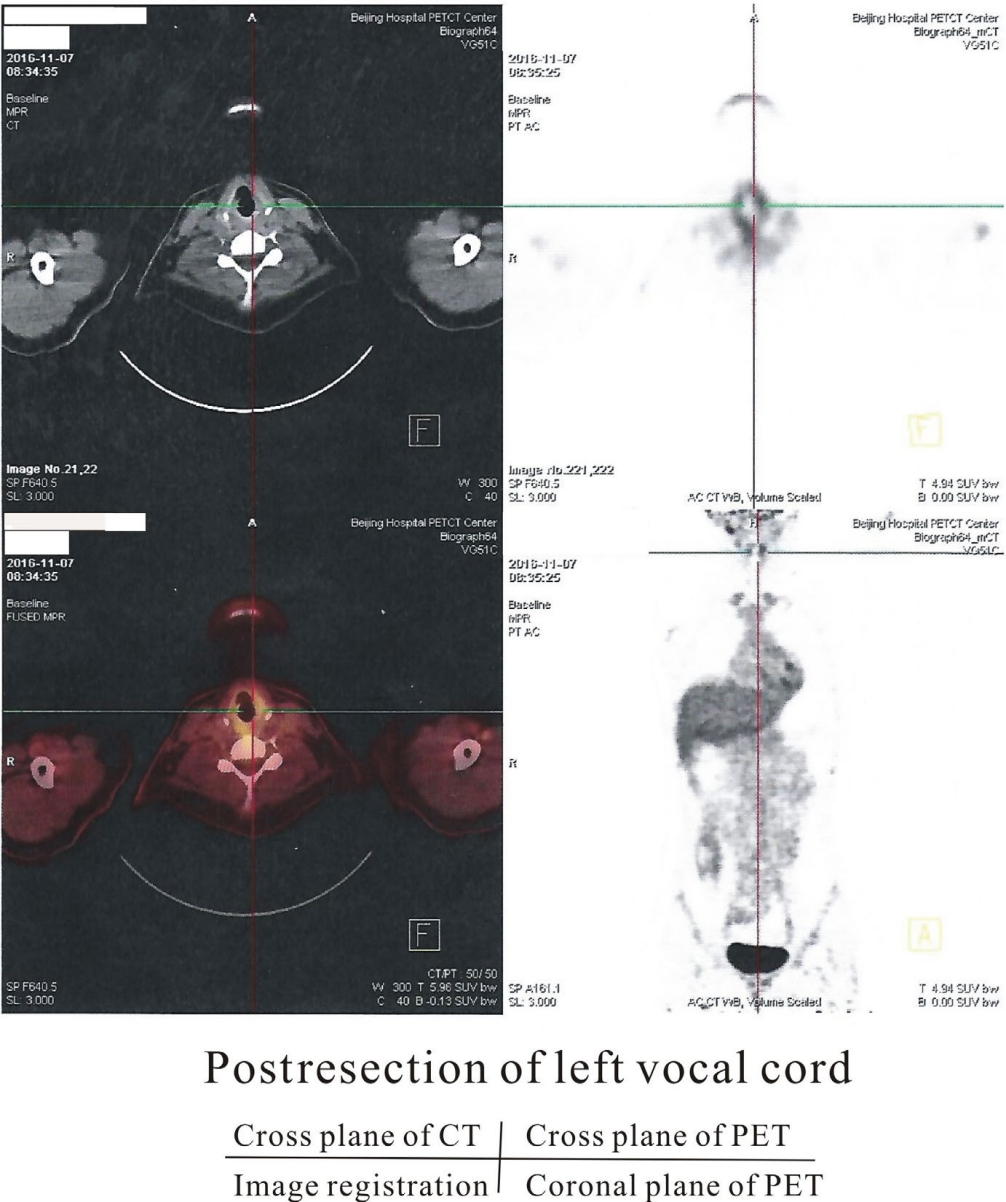
Positron emission tomography/computed tomography examination of the patients after the resection of left vocal cord

To confirm the diagnosis, positive of immunohistochemistry for NUT (nuclear protein in testis) protein was discovered with the help of the PUMCH (Peking Union Medical College Hospital) in November. Though with no obvious evidence of tumor‐related high metabolism in the tissue of tumor bed and the other regions of the body from the results of positron emission tomography/computed tomography (positron emission tomography/computed tomography) (Figure 2), colleagues from PUMCH recommended for subsequent total laryngectomy and neck dissection accompanied with radiotherapy/chemotherapy, in sight of the definitive diagnosis and malignancy of the cancer.

**Figure 3:**
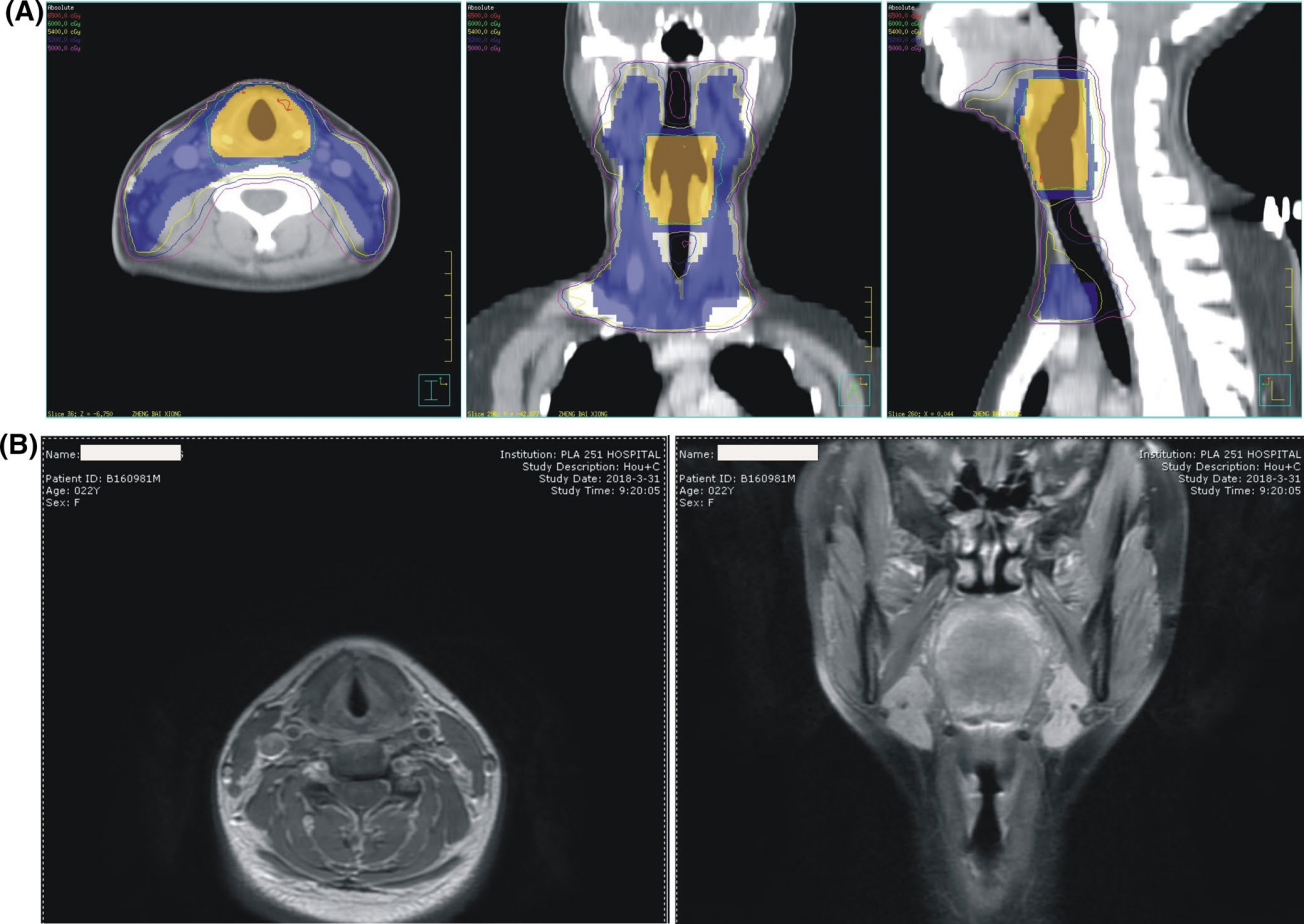
(A) Program of intensity‐modulated radiation therapy; (B) Neck magnetic resonance imaging examination 15 mo after vocal cord resected

Considering the vast cost and some other reasons, she refused their medical advice and then presented at our hospital. Based on her own willingness and medical history, after full examinations, we prescribed the treatment of IMRT (intensity‐modulated radiation therapy) to the dose of 60 Gray (for high‐risk area, 30 fractions) and 54 Gray (for low‐risk area, 30 fractions) (Figure 3A). Meanwhile, administration of Aidi Injection^7^ and Compound Kushen Injection (CKI)^8^ was given to alleviate pain, enhance immunity, and treat tumor. After the radiotherapy (56 Gray/28 f/36 d), with a slight pain in the oropharynx when swallowing and nausea, she went home for recovery. Since then, series of checkup, through enhanced larynx magnetic resonance imaging (Figure 3B) and chest computed tomography, were adopted, and no recurrence in the tumor bed or lymphatic metastasis was detected. Till now, the patient has remained in completely continuous remission for close to 26 months.

## 3 DISCUSSION

Since the first two cases of NMC reported in 1991,^9^, ^10^ the clinic‐pathologic features, treatment characteristics, and prognosis have been widely discussed. NMC is now regarded as a genetically defined neoplasm, considered to be a subtype of squamous cell carcinoma due to presence of histological evidence of abrupt squamous differentiation.^3^, ^11^ Typically, NMC is characterized by chromosomal rearrangements of NUT, at 15q14 and the BRD4 (Bromodomain containing 4) gene, at 19q13, resulting in the t (15; 19) (q14; p13) karyotype.^12^ The BRD‐NUT fusions somehow prevent the expression of genes needed for epithelial differentiation and therefore cause the tumor.^13^ Due to the availability of the antibody to NUT, a protein which is normally exclusive to the testes and diagnostic of NMC for outside expression, the diagnosis of NMC is more convenient and frequent.^14^

However, the laryngeal NMC is still extremely rare with only seven cases reported till now,^3^, ^6^ and we added one new case of our own. There were four males and four females and the mean age of diagnosis is 26.6 years (Table 1), which is in accordance with the young age (avg. age 25) of diagnosis in the original study.^15^ In terms of the clinical characteristics, the reported cases were all located in the supraglottis and in an extensive clinical stage, often Stage Ⅳ. However, our new case was in the left vocal cord with no regional lymph node or distance metastasis, namely in a relatively limited stage.

**Table 1:** Clinicopathological characteristics of laryngeal NMC patients

Author	Age	Sex	Location	Treatment	Died of disease (Months)
Vargas et al^2^	13	F	Epiglottis	QRT + Surgery (Radical neck dissection)	9
Stelow et al^3^	78	F	Supraglottis	QRT + Surgery (Laryngectomy + neck dissection)	8
Kundra et al^4^	39	M	Supraglottis	NA	NA
Henrik Hellquist et al^5^	5	F	Base of tongue	QRT + Surgery (Tracheotomy + bilateral neck dissections + total Laryngectomy for recurrence)	7
Henrik Hellquist et al^5^	41	M	Supraglottis	QRT + Surgery (Partial laryngectomy + neck dissection)	11
Henrik Hellquist et al^5^	17	M	Supraglottis	Surgery (Transoral CO2 Laser Microsurgical resection + neck dissections)	Alive with deasease
Henrik Hellquist et al^5^	47	M	Base of tongue	Palliative chemotherapy	3
Fenghao Geng et al	20	F	Left vocal cord	IMRT + TCM+Surgery (Transoral CO2 Laser Microsurgical resection)	＞26

Abbreviations: NA, not available; QRT, chemoradiotherapy.

Nowadays, there is still no standard treatment for NMC and the clinical outcome for patients with NMC is in general dismal. The impact of surgery on overall survival rate (OS) of NMC patient remains disputed,^2^, ^11^, ^14^ and radiation to a dose of 50 Gray or more with or without chemotherapy is considered to be the primary treatment of NMC in head and neck.^2^ For clinical outcome, the median survival is only 5 months and the 2‐year OS is no more than 10%.^2^, ^14^ Interestingly, all of the three long‐time survivors (＞10 years) of patients with NMC were also those treated with a combination of radiotherapy/chemotherapy.^16^

Based on this study, there may be some explanations responsible for the unusual clinical outcome of our patients and further research is needed for the treatment of patients with NMC.

### 3.1 Whether radiotherapy has the chance to be the first‐line treatment of patients with NMC in limited stage?

Reflecting to the history of the formation of standard treatment for SCLC, we are more confident in challenging the current trend of radical surgery for NMC patients. Study from Fox, W et al,^17^ in 1973 demonstrated that radical radiotherapy provided a better clinical outcome than surgery in the treatment of patients with SCLC in terms of the the mean survival and 10‐year survival. Since then, it has been widely accepted that patients with limited stage SCLC should be administrated with radiotherapy/chemotherapy, instead of radical surgery. And in this case, our patient, at a relatively limited stage, with local resection refused the recommendation from PUMCH for subsequent total laryngectomy and neck dissection accompanied with radiotherapy/chemotherapy and benefited much more from administration with IMRT and TCM (traditional Chinese medicine).

Therefore, considering the benefits of clinical outcome and meanwhile the relief of pain, retention of organ function and improvement of quality of life in this case, we highlight the possibility of radical radiotherapy as the first‐line treatment of NMC patients, especially for those in limited stage. In contrast, radical resection will inevitably cause psychological trauma for those patients.

### 3.2 What matters for the prognosis of NMC patients?

After a series analysis of age, sex, tumor size, regional node involvement, and molecular variants on OS, Prashanth Giridhar et al^2^ ruled out that patients with a larger tumor (＞5 cm) or with regional lymph node metastases had a poorer prognosis. And it was quite a coincidence that tumor size of our patients was only 1 × 1.5 cm and no regional lymph node metastasis was found. In the previous study of the laryngeal NMC patients, clinical outcome was available in six cases, and the survival ranged from 3 to 11 months. Even the most radical treatment failed to save these patients. However, our patient remained in completely continuous remission for close to 26 months. Apart from the different choice of treatment with the reported ones, the relatively early stage should explain for her unusual outcome to some extent.

### 3.3 What else makes a difference for the outcome of our patient?

One the one hand, instead of adopting the radical surgery, we prescribed her with IMRT and TCM after local excision. Our department is one of the former national military centers for integrated Chinese and Western medicine treatment of oncology. Based on our experience and knowledge on TCM, we administrated her with Aidi (80 mL) and CKI (20 mL) for daily injection, both of which are widely accepted to be effective on tumor treatment.

On the other hand, from the initial symptom of hoarseness to definitive diagnosis and treatment, only 5 months were past. We, medical colleagues, and her family struggled together with herself against the dismal cancer for time and fortunately, and we won the battle against laryngeal NMC.

## 4 CONCLUSION

In summary, the present case helps us realize that laryngeal NMC is exceedingly rare but with high malignancy. Radical total resection with the adjuvant radiotherapy/chemotherapy may not be the best choice for those in limited stage. Administration of IMRT and TCM shows the potential for clinical application. Increasing understanding of this rare entity is needed for clinical colleagues and the more efficient to get definitive diagnosis and treatment, the more effective for patients to harbor clinical benefits.

## CONFLICT OF INTEREST

None declared

## AUTHOR CONTRIBUTION

HZ and MHL: prepared the manuscript. SPL involved in IMRT treatment. CBW, ZHZ, YLG, and JYZ: contributed to clinical care for patients and data collection. JZ: revised the paper. FHG: monitored the program.
